# *Delirium* severity in the intensive care unit

**DOI:** 10.62675/2965-2774.20240139-en

**Published:** 2024-10-07

**Authors:** Rodrigo Bernardo Serafim, Maria Carolina Paulino, Tarek Sharshar, Bertrand Hermann

**Affiliations:** 1 Instituto D’Or de Pesquisa e Ensino Rio de Janeiro RJ Brazil Instituto D’Or de Pesquisa e Ensino - Rio de Janeiro (RJ), Brazil.; 2 NOVA Medical School New University of Lisbon Lisbon Portugal NOVA Medical School, New University of Lisbon - Lisbon, Portugal.; 3 Paris-Descartes University Paris France Paris-Descartes University - Paris, France.; 4 Medical School University Paris Cité Paris France Medical School, University Paris Cité - Paris, France.

*Delirium* is a highly prevalent condition in intensive care units (ICUs) and has been associated with poor short- and long-term outcomes. So far, these associations have been demonstrated in studies focusing on *delirium* assessed with binary diagnostic tools (presence or absence of *delirium*). However, recent studies have aimed to identify *delirium* phenotypes, including *delirium* severity, and their associations with patient prognosis. To do so, traditional diagnostic tools have been adapted, and others have been specifically developed to quantify cognitive dysfunction.^(1)^

Quantifying *delirium* can be particularly useful for allocating ICU resources and implementing early preventive or long-term rehabilitation strategies. However, *delirium* severity is a complex concept related to various parameters, including the extent of cognitive impairment, level of arousal, number of *delirium* criteria present, duration of *delirium*, and level of distress experienced by patients.

## Tools for the diagnosis and quantification of delirium severity

The three main instruments used in the ICU to measure *delirium* severity are the Confusion Assessment Method for the ICU (CAM-ICU)-7, which scores each original item from the original CAM-ICU on a scale from 0 to 7 (from 0 to 2: no *delirium*; 3 to 5: mild or moderate *delirium*; and 6 to 7: severe *delirium*);^(1)^the *Delirium* Rating Scale (DRS-R98), which is a longer assessment tool with a *16-item clinician-rated scale with 13 severity items and 3 diagnostic items*;^(2)^and the Intensive Care *Delirium* Screening Checklist (ICDSC), a clinical threshold symptoms scale ranging from 0 to 8 (a higher score associated with greater severity).^(3)^ All these tools are able to characterize *delirium* severity with no clear superiority of one over the other.^(1-3)^

In cases of milder cognitive impairment, in which all diagnostic criteria for *delirium* are not met, the term subsyndromal *delirium* (SSD) is often applied. The ICDSC and CAM-ICU scales have been utilized for SSD diagnosis but lack a well-defined score or consensus, often requiring only one or more positive items without fulfilling all *delirium* criteria.^(4)^

## Subsyndromal delirium

Subsyndromal *delirium* is frequently described as an intermediate stage between *delirium* and a normal mental status not only in terms of presentation but also in terms of the severity of the outcomes. A meta-analysis including 2,630 patients, 36% of whom had SSD, revealed that SSD was associated with a longer hospital length of stay (LOS) than that of *nondelirium* patients.^(4)^ Ouimet et al. described a gradient with increasing ICU LOS for *nondelirium* patients, SSD patients and clinical *delirium* patients (2.5 ± 2.1, 5.2 ± 4.9, and 10.8 ± 11.3 days, respectively; p < 0.01).^(5)^

According to Brummel et al., the duration of SSD in the ICU independently predicts the risk of being institutionalized. Patients with SSD for 5 days are 4.2 times more likely to be institutionalized than those with SSD for 1.5 days.^(6)^ Other studies also revealed that patients with SSD who deteriorated to *delirium* or coma had a longer ICU LOS than those who improved or maintained mental status did (8 [5 - 11] *versus* 6 [4 - 8] days; p = 0.025).^(7)^

## Impact of delirium severity on outcomes

The first studies to discuss that “not all *delirium* is the same” focused on patients’ arousal levels with descriptions of three motoric subtypes (hypoactive, hyperactive and mixed). Since then, there has been substantial evidence that hypoactive *delirium*^(8-10)^ and, to a lesser extent, mixed *delirium* are associated with worse outcomes than hyperactive *delirium* is.^(11,12)^ The worse outcomes in patients with hypoactive *delirium* could be explained by delayed identification.

*Delirium* severity and outcome can also be attributed to presumed etiology, with hypoxic, septic, and sedative-associated *delirium* being associated with worse long-term cognition.^(13)^ However, etiologies often coexist, and the complexity of *delirium* pathophysiology limits the clinical value of these observations. Furthermore, unrecognized subtypes might exist, and unsupervised analyses could help to identify new phenotypes with a better association with outcome.

An intuitive way of assessing *delirium* severity is to quantify the intensity and/or number of *delirium* symptoms, as measured by the CAM-ICU-7, DRS-98 and/or ICDSC. Although the evidence is scarce, greater *delirium* severity seems to be associated with worse outcomes. In a prospective multicentric study, median CAM-ICU-7 scores were independently associated with higher odds of in-hospital mortality (adjusted odds ratio 1.47 [1.30 - 1.66]) and lower odds of being discharged home (adjusted odds ratio 0.8 [0.72 - 0.9]).^(1)^In coronavirus disease 2029 (COVID-19) patients, a greater risk of evolving to coma and mechanical ventilation was found in patients with high mean CAM-ICU-7 scores (scores of 6 to 7), but the risk was not associated with mortality compared with patients with a less severe status.^(14)^ Trajectories of *delirium* severity also seem to correlate with outcomes, with sustained high scores during the first 7 days of admission being associated with higher mortality and longer lengths of stay.^(15)^

In fact, *delirium* duration is the most widely studied measure of *delirium* severity, with a consistent association with mortality and cognitive decline. Several studies reported increased short-term mortality, with a greater duration of *delirium*^(16)^ each day associated with an adjusted hazard ratio for death on the next day of 2.58 [1.32 - 6.21].^(10)^Although less robust, an association with longer-term mortality was found in several studies^(8)^ up to one year.^(17)^ Finally, *delirium* duration is also strongly associated with long-term cognitive decline,^(13)^ with a severity at one year equivalent to mild Alzheimer’s disease or mild traumatic brain injury and a reduced quality of life.^(18)^

The table 1 summarizes the main factors related to the occurrence of delirium and its impact on outcomes.

**Table 1:** Parameters associated with delirium severity and main outcomes

	Parameter	Main outcomes described
*Delirium* severity	Subsyndromal *delirium*	Longer hospital length of stay compared with nondelirium patients^(4)^ Higher risk of institutionalization for subsyndromal *delirium* > 5 days^(6)^
	*Delirium* motoric subtypes (hypoactive, hyperactive or mixed)	Hypoactive delirium has been associated with: Longer *delirium* duration^(11)^ Longer ICU and hospital length of stay^(9)^ Higher mortality^(9)^ Worse 3 and 12 months cognition^(9)^
	Number/intensity of symptoms (high score in assessment tool, CAM-ICU-7, ICDSC, DRS-98)	Greater risk of progression to coma and mechanical ventilation (score of 6 to 7)^(14)^ Longer length of stay^(15)^ Higher mortality^(15)^
	Duration of *delirium*	Higher short-term mortality^(16)^ Higher long-term mortality^(8)^ Long-term cognitive decline^(18)^
	Presumed etiology	Worse long-term cognition in hypoxic, septic, and sedative-associated *delirium*^(13)^

ICU - intensive care unit; CAM-ICU-7 - Confusion Assessment Method for the ICU; ICDSC - Intensive Care Delirium Screening Checklist; DRS-98 - Delirium Rating Scale-Revised-98.

## Conclusion

Routine *delirium* assessment aids in the diagnosis of both full *delirium* and its subsyndromal form, allowing for preventative measures. Severity assessment, either through duration, intensity of symptoms and/or motoric or other subtypes, provides additional prognostic information, potentially allowing for improved management. However, linking severity to outcomes is challenging, and no methods have proven that reducing severity leads to improved outcomes.
