# Supplementary material for: Delirium severity in the intensive care unit
Source: Crit Care Sci. 2024 Oct 7;36:e20240139en. doi: 10.62675/2965-2774.20240139-en (PMC11463989; doi:10.62675/2965-2774.20240139-en)
Supplement: Translation

# Translation: Gravidade do *delirium* na unidade de terapia intensiva

**DOI:** 10.62675/2965-2774.20240139-pt

**Authors:** Rodrigo Bernardo Serafim, Maria Carolina Paulino, Tarek Sharshar, Bertrand Hermann

**Affiliations:** 1 Brasil Instituto D’Or de Pesquisa e Ensino - Rio de Janeiro (RJ), Brasil.; 2 Portugal NOVA Medical School, New University of Lisbon - Lisboa, Portugal.; 3 França Paris-Descartes University - Paris, França.; 4 França Medical School, University Paris Cité - Paris, França.

O *delirium* é uma condição altamente prevalente em unidades de terapia intensiva (UTIs) e tem sido associado a desfechos desfavoráveis em curto e longo prazo. Até o momento, essas associações foram demonstradas em estudos com foco no *delirium* avaliado com ferramentas de diagnóstico binário (presença ou ausência de *delirium*). No entanto, estudos recentes tiveram como objetivo identificar os fenótipos do *delirium*, incluindo a gravidade dele e suas associações com o prognóstico do paciente. Para isso, ferramentas tradicionais de diagnóstico foram adaptadas, e outras foram desenvolvidas especificamente para quantificar a disfunção cognitiva.^(1)^

A quantificação do *delirium* pode ser particularmente útil para alocar recursos na UTI e implementar estratégias preventivas precoces ou de reabilitação em longo prazo. No entanto, a gravidade do *delirium* é um conceito complexo relacionado a vários parâmetros, incluindo a extensão do comprometimento cognitivo, o nível de consciência, o número de critérios de *delirium* presentes, a duração do *delirium* e o nível de estresse experimentado pelos pacientes.

## Ferramentas de diagnóstico e quantificação da gravidade do delirium

Os três principais instrumentos usados na UTI para medir a gravidade do *delirium* são o *Confusion Assessment Method for the ICU* (CAM-ICU)-7, que pontua cada item original do CAM-ICU original em uma escala de zero a sete (de zero a dois: sem *delirium*; três a cinco: *delirium* leve ou moderado; e seis a sete: *delirium* grave);^(1)^ a *Delirium Rating Scale* (DRS-R98), que é uma ferramenta de avaliação mais longa, com uma escala de 16 itens avaliada pelo clínico, com 13 itens de gravidade e três itens de diagnóstico;^(2)^ e a *Intensive Care Delirium Screening Checklist* (ICDSC), uma escala de sintomas de limiar clínico que varia de zero a oito (uma pontuação mais alta associada a maior gravidade).^(3)^ Todas essas ferramentas são capazes de caracterizar a gravidade do *delirium*, sem superioridade clara de uma sobre a outra.^(1-3)^

Em casos de comprometimento cognitivo mais leve, em que não são preenchidos todos os critérios diagnósticos de *delirium*, aplica-se com frequência o termo *delirium* subsindrômico (DSS). A ICDSC e a CAM-ICU têm sido utilizadas para o diagnóstico de DSS, mas carecem de uma pontuação bem definida ou de consenso, muitas vezes exigindo apenas um ou mais itens positivos sem preencher todos os critérios de *delirium*.^(4)^

## Delirium subsindrômico

O DSS é frequentemente descrito como um estágio intermediário entre o *delirium* e um estado mental normal, não apenas em termos de apresentação, mas também em termos da gravidade dos desfechos. Uma metanálise com 2.630 pacientes, 36% dos quais tinham DSS, revelou que o DSS estava associado a uma internação mais longa do que a dos pacientes sem *delirium*.^(4)^ Ouimet et al. descreveram um gradiente com o aumento da internação na UTI de pacientes sem *delirium*, pacientes com DSS e pacientes com *delirium* clínico (2,5 ± 2,1, 5,2 ± 4,9 e 10,8 ± 11,3 dias, respectivamente; p < 0,01).^(5)^

De acordo com Brummel et al., a duração do DSS na UTI prevê de forma independente o risco de ser institucionalizado. Pacientes com DSS por 5 dias têm 4,2 vezes mais probabilidade de serem institucionalizados do que aqueles com DSS por 1,5 dia.^(6)^ Outros estudos também revelaram que pacientes com DSS que se deterioraram para *delirium* ou coma tiveram internação mais longa na UTI do que aqueles que melhoraram ou mantiveram o estado mental (8 [5 - 11] *versus* 6 [4 - 8] dias; p = 0,025).^(7)^

## Impacto da gravidade do delirium nos desfechos

Os primeiros estudos a discutir que “nem todo *delirium* é igual” se concentraram nas alterações do estado mental dos pacientes com descrições de três subtipos motores (hipoativo, hiperativo e misto). Desde então, há evidências substanciais de que o *delirium* hipoativo^(8-10)^ e, em menor grau, o misto estão associados a desfechos mais desfavoráveis do que o hiperativo.^(11,12)^ Os desfechos mais desfavoráveis em pacientes com *delirium* hipoativo podem ser explicados pela identificação tardia.

A gravidade e o desfecho do *delirium* também podem ser atribuídos à etiologia presumida, sendo que o *delirium* hipóxico, séptico e associado a sedativos está ligado a um declínio cognitivo em longo prazo.^(13)^ Entretanto, as etiologias frequentemente coexistem, e a complexidade da fisiopatologia do *delirium* limita o valor clínico dessas observações. Além disso, podem existir subtipos não reconhecidos, e análises não supervisionadas poderiam ajudar a identificar novos fenótipos com uma melhor associação com o desfecho.

Uma forma intuitiva de avaliar a gravidade do *delirium* é quantificar a intensidade e/ou o número de sintomas de *delirium*, conforme medido pelo CAM-ICU-7, DRS-98 e/ou ICSDC. Embora as evidências sejam escassas, a maior gravidade do *delirium* parece estar associada a desfechos desfavoráveis. Em um estudo prospectivo multicêntrico, as pontuações medianas do CAM-ICU-7 foram associadas de forma independente a maiores chances de mortalidade hospitalar (razão de chances ajustada 1,47 [1,30 - 1,66]) e menores chances de receber alta para casa (razão de chances ajustada 0,8 [0,72 - 0,9]).^(1)^ Em pacientes com doença pelo coronavírus 2019 (COVID-19), um risco maior de evoluir a coma e à ventilação mecânica foi encontrado em pacientes com pontuações médias altas de CAM-ICU-7 (pontuações de 6 a 7), mas o risco não foi associado à mortalidade em comparação com pacientes em estado menos grave.^(14)^ As trajetórias de gravidade do *delirium* também parecem se correlacionar com os desfechos, com pontuações altas sustentadas durante os primeiros 7 dias de admissão sendo associadas à maior mortalidade e a períodos mais longos de internação.^(15)^

De fato, a duração do *delirium* é a medida mais amplamente estudada da gravidade do *delirium*, com uma associação consistente com mortalidade e declínio cognitivo. Vários estudos relataram um aumento da mortalidade em curto prazo, com maior duração do *delirium*^(16)^ a cada dia associado a uma razão de risco ajustada de morte no dia seguinte de 2,58 [1,32 - 6,21].^(10)^ Embora menos robusta, uma associação com mortalidade em longo prazo foi encontrada em vários estudos^(8)^ até 1 ano.^(17)^ Por fim, a duração do *delirium* também está fortemente associada ao declínio cognitivo em longo prazo,^(13)^ com gravidade em 1 ano equivalente à da doença de Alzheimer leve ou da lesão cerebral traumática leve e diminuição da qualidade de vida.^(18)^

A tabela 1 resume os principais fatores relacionados à ocorrência do *delirium* e seu impacto nos desfechos.

**Tabela 1:** Parâmetros associados à gravidade do *delirium* e aos principais desfechos

	Parâmetros	Principais desfechos descritos
Gravidade do *delirium*	*Delirium* subsindrômico	Internações hospitalares mais longas em comparação com pacientes sem *delirium*^(4)^ Maior risco de institucionalização em caso de *delirium* subsindrômico > 5 dias^(6)^
	Subtipos motores de *delirium* (hipoativo, hiperativo ou misto)	O *delirium* hipoativo tem sido associado a: *Delirium* mais prolongado^(11)^ Internação mais longa na UTI e no hospital^(9)^ Maior mortalidade^(9)^ Declínio cognitivo em 3 e 12 meses^(9)^
	Número/intensidade dos sintomas (pontuação alta na ferramenta de avaliação, CAM-ICU-7, ICDSC, DRS-98)	Maior risco de progressão para coma e ventilação mecânica (pontuação de 6 a 7)^(14)^ Internação mais longa^(15)^ Maior mortalidade^(15)^
	Duração do *delirium*	Maior mortalidade em curto prazo^(16)^ Maior mortalidade em longo prazo^(8)^ Declínio cognitivo em longo prazo^(18)^
	Etiologia presumida	Declínio cognitivo em longo prazo no *delirium* associado a hipóxia, septicemia e sedativos^(13)^

UTI - unidade de terapia intensiva; CAM-ICU-7 - *Confusion Assessment Method for the ICU*; ICDSC - *Intensive Care Delirium Screening Checklist*; DRS-98 - *Delirium Rating Scale-Revised-*98.

## Conclusão

A avaliação periódica do *delirium* auxilia no diagnóstico tanto do *delirium* propriamente dito, quanto de sua forma subsindrômica, permitindo medidas preventivas. A avaliação da gravidade, seja por meio da duração, da intensidade dos sintomas e/ou dos subtipos motores ou outros, fornece maiores informações prognósticas, possibilitando, provavelmente, um manejo melhor. No entanto, relacionar a gravidade aos desfechos é desafiador, e nenhum método concluiu que a redução da gravidade resulta em desfechos melhores.
